# Supplementary material for: Early identification of postpartum depression using demographic, clinical, and digital phenotyping
Source: Transl Psychiatry. 2021 Feb 11;11:121. doi: 10.1038/s41398-021-01245-6 (PMC7878890; doi:10.1038/s41398-021-01245-6)
Supplement: Supplementary file 1 — Supplement 1 [file 41398_2021_1245_MOESM1_ESM.docx]

Hahn et al.

**Early identification of postpartum depression using demographic, clinical, and digital phenotyping**

**Supplementary materials**

*Exclusion criteria*

Exclusion criteria were based either on the maternal psychiatric status or on the child’s condition and expected outcome. Exclusion criteria based on the maternal psychiatric status were: 1) alcoholic or psychotic substance dependency or use during current pregnancy, 2) antidepressant or antipsychotic medication during pregnancy, 3) history of psychosis or manic episodes, 4) current depressive episode at the time of recruitment, and 5) lack of sufficient understanding of German or English. Exclusion criteria based on the child’s condition and expected outcome were: 1) genetic defects (e.g. trisomy), premature birth (i.e. less than 29 weeks of gestation) and very low weight (<1000g), pathological neurological assessment based on the German Child Health tests (U2) within one to six days after birth.

| *Table S1: Sociodemographic-anamnestic data in the first and validation cohort.* | | | | | | | | |
| --- | --- | --- | --- | --- | --- | --- | --- | --- |
|  | **1^st^ cohort** | | | | **Validation cohort** | | | |
| **Sociodemographic- anamnestic variable** | **HC** | **PPD** | **AD** | **Statistical test** | **HC** | **PPD** | **AD** | **Statistical test** |
| Family status (no partner/partner) | 4/242 | 4/24 | 0/33 | **°** | 1/144 | 2/14 | 1/29 | **°** |
| Marital status (unmarried/ married) | 62/184 | 13/15 | 9/24 | X²(2, N= 307) = 5.70  p = .058 | 37/108 | 7/9 | 8/22 | X²(2, N= 191) = 2.42  p = .30 |
| Total number of children | 1.63 ± 0.78  N = 246 | 1.74 ± 0.90  N = 27 | 1.42 ± 0.71  N = 33 | X²(2, N= 306) = 4.50  p = .11 | 1.68 ± 0.84  N = 146 | 1.75 ± 0.86  N = 16 | 1.67 ± 0.99  N = 30 | X²(2, N= 192) = 0.10  p = .95 |
| Completed professional education (no/ yes) | 24/222 | 6/22 | 5/28 | **°** | 8/137 | 3/13 | 2/26 | **°** |
| Annual income (<10,000€/ <20,000€/≤50,000€/>50,000€) | 3/28/78/127 | 3/5/11/8 | 0/0/17/16 | **°** | 0/9/45/85 | 1/4/5/5 | 0/0/15/12 | **°** |
| Psychiatric diagnosis in previous pregnancy (no/ yes) | 238/8 | 22/5 | 31/2 | **°** | 141/2 | 11/5 | 27/3 | **°** |
| Week of gestation  (in days) | 273 ± 12.4  N = 247 | 269 ± 19.3  N = 28 | 272 ± 15.0  N = 33 | X²(2, N= 308) = 1.68  p = .43 | 274 ± 11.2  N = 145 | 275 ± 10.4  N = 16 | 267 ± 22.3  N = 30 | X²(2, N= 191) = 6.69  p = .035 ***^2,3^** |
| Child’s gender (male/female/ both(twins)) | 119/125/2 | 14/14/0 | 18/15/0 | X²(2, N= 307) = 0.89  p = .93 | 69/77/1 | 9/5/2 | 14/16/0 | **°** |
| Child’s birth weight (in gram) | 3340 ± 534  N = 237 | 3286 ± 730  N = 28 | 3211 ± 697  N = 31 | X²(2, N= 296) = 1.47  p = .48 | 3306 ± 496  N = 147 | 3108 ± 368  N = 16 | 3035 ± 808  N = 30 | X²(2, N= 193) = 6.77  p = .034 ***^1,2^** |
| Relocation of the child to neonatal ward (no/yes) | 182/64 | 19/9 | 19/14 | X²(2, N= 307) = 4.08  p = .13 | 110/35 | 10/6 | 18/12 | X²(2, N= 191) = 3.95  p = .14 |
| Breastfeeding T0 (no/yes) | 28/219 | 6/22 | 4/29 | **°** | 19/125 | 1/15 | 1/29 | **°** |
| Quality of support at home (insufficient/ sufficient) | 2/165 | 5/17 | 1/25 | **°** | 138/0 | 15/1 | 28/1 | **°** |
| **°** No statistical analysis possible due to low expected cell counts.  * Bonferroni-corrected significant difference (p < .05) between **^1^** HC and PPD, **^2^** between HC and AD and/or **^3^** between PPD and AD  ■ Significant group difference (p < .05) between first and validation cohort | | | | | | | | |


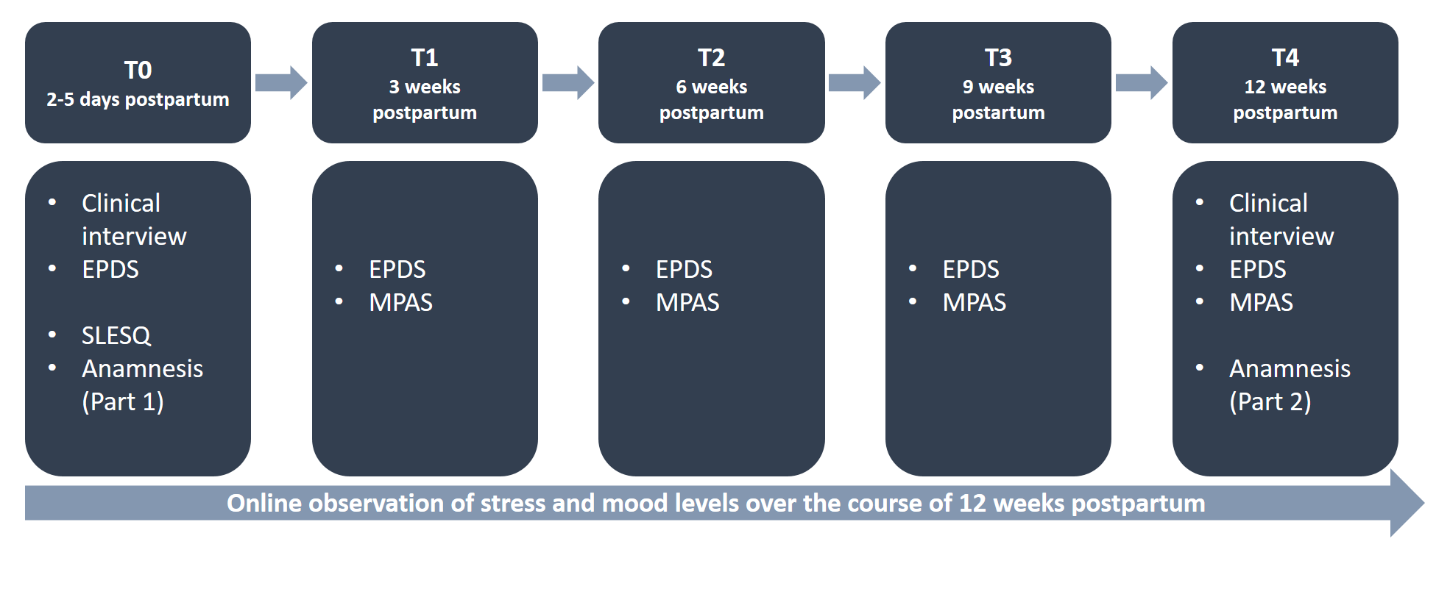


**Figure S1**. Study design. EPDS: Edinburgh Postnatal Depression Scale. SLESQ: Stressful Life Events Screening Questionnaire. MPAS: Maternal Postnatal Attachment Scale.

| *Table S2:* βX combinations for the logistic regression class probability formula for validation analyses | |
| --- | --- |
| Group | βX |
| HC vs. PPD | 2.014 - 0.123 * baseline EPDS – 0.56 * EPDS at week 3 + 0.711 * mood at week 3 |
| HC vs. AD | - 13.31 – 0.296 * baseline EPDS – 1.162* EPDS at week 3 + 0.268 * MPAS at week 3 + 0.716 * mood at week 3 |
| PPD vs. AD | - 4.146 + 0.658 * mood at week 6 |
| AD – adjustment disorder, HC – healthy controls, PPD – postpartum depression | |

| *Table S3: Odds ratios for the first and validation cohort.* | | | | | | |
| --- | --- | --- | --- | --- | --- | --- |
|  | **first cohort** | | | **validation cohort** | | |
| **Sociodemographic-anamnestic variable** | **Odds ratio**  **(95 % Confidence interval)** | | | **Odds ratio**  **(95 % Confidence interval)** | | |
|  | **HC vs. PPD** | **HC vs. AD** | **PPD vs. AD** | **HC vs. PPD** | **HC vs. AD** | **PPD vs. AD** |
| Age (in years) |  | -- |  |  | -- |  |
| Family status (no partner/partner) | OR = 10.1  (8.64, 11.5) | -- | -- | OR = 20.6  (18.1, 23.0) | OR = 4.97  (2.17, 7.77) | OR = 4.14  (1.66, 6.63) |
| Marital status (unmarried/married) | OR = 2.57  (1.78, 3.37) | OR = 1.11  (0.29, 1.93) | OR = 2.31  (1.24, 3.38) | OR = 2.27  (1.21, 3.33) | OR = 1.06  (0.17, 1.95) | OR = 2.14  (0.86, 3.42) |
| Number of children |  | -- |  |  | -- |  |
| Education (in years) |  | -- |  |  | -- |  |
| Completed professional education (no/yes) | OR = 2.52  (1.53, 3.52) | OR = 1.65  (0.61, 2.69) | OR = 1.53  (0.22, 2.84) | OR = 3.95  (2.51, 5.40) | OR = 1.32  (-0.29, 2.92) | OR = 3.00  (1.09, 4.91) |
| Annual income (<10,000€/<20,000€/ ≤50,000€/>50,000€) |  | -- |  |  | **--** |  |
| Personal psychiatric history (no/yes) | OR = 6.61  (5.26, 6.96) | OR = 6.00  (5.21, 6.80) | OR = 1.02  (0.00, 2.04) | OR = 7.56  (6.47, 8.66) | OR = 4.54  (3.71, 5.37) | OR = 1.67  (0.43, 2.91) |
| Familial psychiatric history (no/yes) | OR = 2.75  (1.94, 3.55) | OR = 3.05  (2.30, 3.80) | OR = 0.90  (-0.11, 1.91) | OR = 3.39  (2.34, 4.45) | OR = 2.97  (2.15, 3.79) | OR = 1.14  (-0.072, 2.36) |
| Psychiatric diagnosis in previous pregnancy (no/yes) | OR = 6.76  (5.56, 7.96) | OR = 1.92  (0.33, 3.51) | OR = 3.52  (1.79, 5.25) | OR = 32.1  (30.3, 33.8) | OR = 7.83  (6.00, 9.67) | OR = 4.09  (2.50, 5.68) |
| Birth complications (no/yes) | OR = 2.26  (1.37, 3.15) | OR = 2.12  (1.28, 2.96) | OR = 1.07  (-0.06, 2.19) | OR = 2.79  (1.70, 3.89) | OR = 0.93  (-0.12, 1.98) | OR = 3.00  (0.86, 3.74) |
| Week of gestation  (in days) |  | -- |  |  | -- |  |
| Subjective birth-related psychological traumas (no/yes) | OR = 3.51  (2.63, 4.39) | OR = 4.82  (4.02, 5.62) | OR = 0.73  (-0.33, 1.79) | -- | OR = 2.70  (1.69, 3.71) | OR = 1.10  (-0.32, 2.51) |
| Child’s gender (male/female/both(twins)) | OR = 0.95  (0.17, 1.73) | OR = 0.79  (0.06, 1.52) | OR = 1.20  (0.19, 2.21) | OR = 0.50  (-0.64, 1.64) | OR = 1.02  (0.24, 1.81) | OR = 0.49  (-0.82, 1.79) |
| Child’s birth weight (in gram) |  | -- |  |  | -- |  |
| Child relocated to another ward (no/yes) | OR = 1.35  (0.50, 2.19) | OR = 2.10  (1.35, 2.84) | OR = 0.64  (-0.41, 1.69) | OR = 1.89  (0.80, 2.97) | OR = 2.10  (1.27, 2.92) | OR = 0.90  (-0.35, 2.15) |
| Breastfeeding T0 (no/yes) | OR = 2.13  (1.15, 3.12) | OR = 1.08  (-0.04, 2.20) | OR = 1.98  (0.60, 3.36) | OR = 0.44  (-1.64, 2.52) | OR = 0.23  (-1.82, 2.28) | OR = 1.93  (-0.91, 4.77) |
| PMS (no PMS/mild PMS/PMS) |  | -- |  |  | -- |  |
| Postpartum blues (no/yes) | OR = 4.06  (3.20, 4.92) | OR = 6.03  (5.16, 6.90) | OR = 0.67  (-0.50, 1.84) | -- | OR = 5.29  (4.43, 6.14) | -- |
| Stressful life events (no/yes) | OR = 2.16  (1.36, 2.96) | OR = 2.45  (1.69, 3.20) | OR = 0.88  (-0.16, 1.92) | OR = 6.72  (5.20, 8.23) | OR = 2.64  (1.77, 3.51) | OR = 2.55  (0.86, 4.23) |
| Quality of support at home (insufficient/sufficient) | OR = 0.04  (-1.67, 1.76) | OR = 0.30  (-2.13, 2.74) | OR = 0.14  (-2.10, 2.37) | -- | -- | OR = 0.53  (-2.31, 3.38) |
| Breastfeeding T4 (no/yes) | OR = 2.89  (2.09, 3.68) | OR = 0.92  (0.08, 1.77) | OR = 3.13  (2.04, 4.21) | OR = 3.01  (1.96, 4.06) | OR = 1.05  (0.19, 1.92) | OR = 2.86  (1.59, 4.12) |

| *Table S4: Post-hoc analyses for mood in the first cohort. Bonferroni-corrected p-values.* | | | | | | | |
| --- | --- | --- | --- | --- | --- | --- | --- |
| Week | Group | N | Mean | SD | HC | AD |  |
| Week 0 | HC | 242 | 9.08 | 1.48 |  |  |  |
|  | AD | 28 | 7.91 | 2.34 | p = .001 * |  |  |
|  | PPD | 33 | 8.29 | 2.27 | p = .055 | p = 1.00 |  |
| Week 1 | HC | 247 | 8.44 | 1.44 |  |  |  |
|  | AD | 28 | 6.11 | 2.05 | p < .001 ** |  |  |
|  | PPD | 33 | 6.71 | 1.83 | p < .001 ** | p = .395 |  |
| Week 2 | HC | 247 | 8.32 | 1.48 |  |  |  |
|  | AD | 28 | 5.95 | 1.59 | p < .001 ** |  |  |
|  | PPD | 33 | 6.20 | 1.98 | p < .001 ** | p = 1.00 |  |
| Week 3 | HC | 246 | 8.37 | 1.42 |  |  |  |
|  | AD | 28 | 6.03 | 1.69 | p < .001 ** |  |  |
|  | PPD | 33 | 6.08 | 1.89 | p < .001 ** | p = .468 |  |
| Week 4 | HC | 245 | 8.35 | 1.42 |  |  |  |
|  | AD | 28 | 7.14 | 1.67 | p < .001 ** |  |  |
|  | PPD | 33 | 5.85 | 2.03 | p < .001 ** | p = .003 * |  |
| Week 5 | HC | 246 | 8.36 | 1.51 |  |  |  |
|  | AD | 28 | 7.19 | 1.77 | p < .001 ** |  |  |
|  | PPD | 33 | 5.67 | 2.25 | p < .001 ** | p = .001 * |  |
| Week 6 | HC | 245 | 8.41 | 1.47 |  |  |  |
|  | AD | 28 | 7.51 | 1.52 | p = .004 * |  |  |
|  | PPD | 33 | 5.58 | 1.96 | p < .001 ** | p < .001 ** |  |
| Week 7 | HC | 246 | 8.37 | 1.49 |  |  |  |
|  | AD | 28 | 7.87 | 1.30 | p = .241 |  |  |
|  | PPD | 33 | 5.66 | 1.49 | p < .001 ** | p < .001 ** |  |
| Week 8 | HC | 246 | 8.44 | 1.49 |  |  |  |
|  | AD | 28 | 8.04 | 1.38 | p = .514 |  |  |
|  | PPD | 33 | 5.26 | 2.21 | p < .001 ** | p < .001 ** |  |
| Week 9 | HC | 244 | 8.41 | 1.47 |  |  |  |
|  | AD | 28 | 8.29 | 1.27 | p = 1.00 |  |  |
|  | PPD | 33 | 5.25 | 2.32 | p < .001 ** | p < .001 ** |  |
| Week 10 | HC | 246 | 8.52 | 1.45 |  |  |  |
|  | AD | 28 | 8.29 | 1.15 | p = 1.00 |  |  |
|  | PPD | 33 | 5.28 | 1.90 | p < .001 ** | p < .001 ** |  |
| Week 11 | HC | 242 | 8.51 | 1.47 |  |  |  |
|  | AD | 28 | 8.35 | 1.21 | p = 1.00 |  |  |
|  | PPD | 32 | 4.67 | 1.75 | p < .001 ** | p < .001 ** |  |
| Week 12 | HC | 155 | 8.59 | 1.60 |  |  |  |
|  | AD | 24 | 8.50 | 1.14 | p = 1.00 |  |  |
|  | PPD | 33 | 4.10 | 1.96 | p < .001 ** | p < .001 ** |  |
| *significant at p < .05  ** significant at p < .001 | | | | | | |  |

| *Table S5: Post-hoc analyses for mood-stress difference in the first cohort.* Bonferroni-corrected p-values. | | | | | | |
| --- | --- | --- | --- | --- | --- | --- |
| Week | Group | N | Mean | SD | HC | AD |
| Week 0 | HC | 240 | 0.01 | 1.63 |  |  |
|  | AD | 27 | -1.00 | 1.81 | p = .003* |  |
|  | PPD | 33 | -0.21 | 2.02 | p = 1.00 | p = .206 |
| Week 1 | HC | 246 | 0.07 | 1.29 |  |  |
|  | AD | 28 | -1.95 | 1.63 | p < .001 ** |  |
|  | PPD | 33 | -1.19 | 1.34 | p < .001 ** | p = .076 |
| Week 2 | HC | 247 | 0.08 | 1.26 |  |  |
|  | AD | 28 | -1.87 | 1.37 | p < .001 ** |  |
|  | PPD | 33 | -1.67 | 1.09 | p < .001 ** | p = 1.00 |
| Week 3 | HC | 246 | 0.16 | 1.20 |  |  |
|  | AD | 28 | -1.21 | 1.27 | p < .001 ** |  |
|  | PPD | 33 | -1.60 | 1.08 | p < .001 ** | p = .616 |
| Week 4 | HC | 245 | 0.22 | 1.27 |  |  |
|  | AD | 28 | -0.79 | 1.42 | p < .001 ** |  |
|  | PPD | 33 | -1.88 | 1.20 | p < .001 ** | p = .003 * |
| Week 5 | HC | 246 | 0.27 | 1.24 |  |  |
|  | AD | 28 | -0.47 | 1.54 | p = .005 * |  |
|  | PPD | 33 | -1.87 | 1.57 | p < .001 ** | p < .001 ** |
| Week 6 | HC | 245 | 0.32 | 1.25 |  |  |
|  | AD | 28 | -0.33 | 1.30 | p = .012 * |  |
|  | PPD | 33 | -1.92 | 1.45 | p < .001 ** | p < .001 ** |
| Week 7 | HC | 246 | 0.31 | 1.27 |  |  |
|  | AD | 28 | 0.05 | 1.08 | p = .679 |  |
|  | PPD | 33 | -1.89 | 1.61 | p < .001 ** | p < .001 ** |
| Week 8 | HC | 245 | 0.39 | 1.29 |  |  |
|  | AD | 28 | 0.09 | 1.11 | p = .558 |  |
|  | PPD | 33 | -2.19 | 1.87 | p < .001 ** | p < .001 ** |
| Week 9 | HC | 244 | 0.41 | 1.26 |  |  |
|  | AD | 28 | 0.35 | 1.05 | p = 1.00 |  |
|  | PPD | 33 | -2.18 | 1.60 | p < .001 ** | p < .001 ** |
| Week 10 | HC | 246 | 0.55 | 1.22 |  |  |
|  | AD | 28 | 0.40 | 1.05 | p = 1.00 |  |
|  | PPD | 33 | -2.39 | 1.40 | p < .001 ** | p < .001 ** |
| Week 11 | HC | 242 | 0.54 | 1.26 |  |  |
|  | AD | 28 | 0.50 | 1.15 | p = 1.00 |  |
|  | PPD | 32 | -2.91 | 1.21 | p < .001 ** | p < .001 ** |
| Week 12 | HC | 149 | 0.57 | 1.38 |  |  |
|  | AD | 21 | 0.82 | 1.05 | p = .544 |  |
|  | PPD | 22 | -3.03 | 1.61 | p < .001 ** | p < .001 ** |
| *significant at p < .05  ** significant at p < .001 | | | | | | |

| *Table S6: Post-hoc analyses for stress in the first cohort.* Bonferroni-corrected p-values. | | | | | | |
| --- | --- | --- | --- | --- | --- | --- |
| Week | Group | N | Mean | SD | HC | AD |
| Week 0 | HC | 240 | 5.17 | 2.92 |  |  |
|  | AD | 27 | 6.18 | 2.84 | p = .179 |  |
|  | PPD | 33 | 4.81 | 2.65 | p = 1.00 | p = .209 |
| Week 1 | HC | 246 | 4.27 | 1.97 |  |  |
|  | AD | 28 | 6.24 | 2.08 | p < .001 ** |  |
|  | PPD | 33 | 5.20 | 1.91 | p = .058 | p = .121 |
| Week 2 | HC | 247 | 4.11 | 1.81 |  |  |
|  | AD | 28 | 5.87 | 2.05 | p < .001 ** |  |
|  | PPD | 33 | 5.69 | 1.84 | p < .001 ** | p = 1.00 |
| Week 3 | HC | 247 | 3.97 | 1.81 |  |  |
|  | AD | 28 | 5.15 | 1.87 | p = .002 * |  |
|  | PPD | 33 | 5.40 | 1.77 | p < .001 ** | p = 1.00 |
| Week 4 | HC | 246 | 3.81 | 1.86 |  |  |
|  | AD | 28 | 4.78 | 1.98 | p = .017 * |  |
|  | PPD | 33 | 5.77 | 1.84 | p < .001 ** | p = .117 |
| Week 5 | HC | 247 | 3.72 | 1.78 |  |  |
|  | AD | 28 | 4.11 | 1.86 | p = .708 |  |
|  | PPD | 33 | 5.51 | 1.98 | p < .001 ** | p = .009 * |
| Week 6 | HC | 246 | 3.66 | 1.76 |  |  |
|  | AD | 28 | 4.15 | 1.61 | p = .401 |  |
|  | PPD | 33 | 5.54 | 2.02 | p < .001 ** | p = .008 * |
| Week 7 | HC | 247 | 3.64 | 1.86 |  |  |
|  | AD | 28 | 3.72 | 1.52 | p = 1.00 |  |
|  | PPD | 33 | 5.56 | 2.16 | p < .001 ** | p < .001 ** |
| Week 8 | HC | 246 | 3.55 | 1.84 |  |  |
|  | AD | 28 | 3.83 | 1.70 | p = 1.00 |  |
|  | PPD | 33 | 5.78 | 2.18 | p < .001 ** | p < .001 ** |
| Week 9 | HC | 245 | 3.44 | 1.79 |  |  |
|  | AD | 28 | 3.52 | 1.52 | p = 1.00 |  |
|  | PPD | 33 | 5.75 | 1.86 | p < .001 ** | p < .001 ** |
| Week 10 | HC | 246 | 3.28 | 1.78 |  |  |
|  | AD | 28 | 3.42 | 1.50 | p = 1.00 |  |
|  | PPD | 33 | 6.24 | 1.88 | p < .001 ** | p < .001 ** |
| Week 11 | HC | 242 | 3.28 | 1.73 |  |  |
|  | AD | 28 | 3.25 | 1.59 | p = 1.00 |  |
|  | PPD | 32 | 6.74 | 1.99 | p < .001 ** | p < .001 ** |
| Week 12 | HC | 151 | 3.26 | 1.90 |  |  |
|  | AD | 22 | 2.66 | 1.35 | p = .511 |  |
|  | PPD | 22 | 6.61 | 2.56 | p < .001 ** | p < .001 ** |
| *significant at p < .05  ** significant at p < .001 | | | | | | |

| *Table S7: Balanced accuracies for predictive modeling incl. baseline and follow-up EPDS scores.* | | | | | | | | | | |
| --- | --- | --- | --- | --- | --- | --- | --- | --- | --- | --- |
| Week | Classifier | Feature Combination | | | | | | | | |
|  |  | 1 | 2 | 3 | 4 | 5 | 6 | 7 | 8 | 9 |
| 0 | HC vs. PPD | 0.7683 | 0.7613 | 0.7667 | 0.7582 | 0.7606 | 0.7665 | 0.7588 | 0.7238 | 0.7242 |
|  | HC vs. AD | 0.7710 | 0.7580 | 0.7583 | 0.7507 | 0.7576 | 0.7585 | 0.7503 | 0.7494 | 0.7499 |
|  | PPD vs. AD | 0.4999 | 0.5012 | 0.5943 | 0.5633 | 0.4978 | 0.5969 | 0.5637 | 0.5741 | 0.5705 |
| 1 | HC vs. PPD | 0.7684 | 0.7685 | 0.7665 | 0.7585 | 0.7629 | 0.7755 | 0.7574 | 0.7493 | 0.7481 |
|  | HC vs. AD | 0.7704 | 0.8024 | 0.7733 | 0.7927 | 0.7964 | 0.7657 | 0.7857 | 0.7896 | 0.7909 |
|  | PPD vs. AD | 0.4997 | 0.5116 | 0.5850 | 0.6464 | 0.4997 | 0.5757 | 0.6181 | 0.5511 | 0.5501 |
| 2 | HC vs. PPD | 0.7690 | 0.7579 | 0.7507 | 0.7846 | 0.7532 | 0.7306 | 0.7731 | 0.7534 | 0.7531 |
|  | HC vs. AD | 0.7710 | 0.8019 | 0.7502 | 0.7814 | 0.7944 | 0.7527 | 0.7846 | 0.7732 | 0.7749 |
|  | PPD vs. AD | 0.4998 | 0.4800 | 0.4665 | 0.4675 | 0.4745 | 0.5464 | 0.5612 | 0.5020 | 0.5002 |
| 3 | HC vs. PPD | 0.8211 | 0.8681 | 0.8232 | 0.8478 | 0.8626 | 0.8008 | 0.8182 | 0.8205 | 0.8218 |
|  | HC vs. AD | 0.8898 | 0.8993 | 0.8886 | 0.8945 | 0.8972 | 0.8839 | 0.8868 | 0.8786 | 0.8792 |
|  | PPD vs. AD | 0.4983 | 0.4836 | 0.4841 | 0.5415 | 0.5545 | 0.5388 | 0.5925 | 0.5498 | 0.5512 |
| 4 | HC vs. PPD | 0.8222 | 0.8602 | 0.8388 | 0.8490 | 0.8650 | 0.8254 | 0.8333 | 0.8346 | 0.8343 |
|  | HC vs. AD | 0.8896 | 0.9039 | 0.8871 | 0.8899 | 0.8892 | 0.8761 | 0.8804 | 0.8729 | 0.8732 |
|  | PPD vs. AD | 0.4989 | 0.5947 | 0.6464 | 0.6678 | 0.6008 | 0.6103 | 0.6334 | 0.5948 | 0.5955 |
| 5 | HC vs. PPD | 0.8219 | 0.8359 | 0.8218 | 0.8283 | 0.8398 | 0.8193 | 0.8342 | 0.8217 | 0.8221 |
|  | HC vs. AD | 0.8900 | 0.8979 | 0.8913 | 0.8916 | 0.8897 | 0.8774 | 0.8776 | 0.8768 | 0.8759 |
|  | PPD vs. AD | 0.4991 | 0.6170 | 0.6143 | 0.6655 | 0.5966 | 0.5912 | 0.6489 | 0.5822 | 0.5869 |
| 6 | HC vs. PPD | 0.9077 | 0.8918 | 0.8839 | 0.8826 | 0.8810 | 0.8794 | 0.8792 | 0.8610 | 0.8610 |
|  | HC vs. AD | 0.8988 | 0.8989 | 0.9140 | 0.8950 | 0.8855 | 0.9050 | 0.8801 | 0.8919 | 0.8909 |
|  | PPD vs. AD | 0.7106 | 0.7331 | 0.6995 | 0.7356 | 0.7298 | 0.7216 | 0.7443 | 0.6952 | 0.6993 |
| 7 | HC vs. PPD | 0.9079 | 0.9059 | 0.8979 | 0.8968 | 0.8816 | 0.8726 | 0.8754 | 0.8678 | 0.8668 |
|  | HC vs. AD | 0.8994 | 0.8985 | 0.9152 | 0.8996 | 0.8866 | 0.9085 | 0.8813 | 0.8946 | 0.8933 |
|  | PPD vs. AD | 0.7110 | 0.7304 | 0.7317 | 0.7256 | 0.7183 | 0.7192 | 0.7339 | 0.6970 | 0.6909 |
| 8 | HC vs. PPD | 0.9073 | 0.8969 | 0.9021 | 0.8954 | 0.8768 | 0.8737 | 0.8702 | 0.8461 | 0.8481 |
|  | HC vs. AD | 0.8985 | 0.8956 | 0.9050 | 0.9033 | 0.8948 | 0.9091 | 0.8920 | 0.8940 | 0.8942 |
|  | PPD vs. AD | 0.7112 | 0.7710 | 0.7601 | 0.7876 | 0.7344 | 0.7232 | 0.7555 | 0.7124 | 0.7162 |
| 9 | HC vs. PPD | 0.9118 | 0.9045 | 0.9014 | 0.9013 | 0.8977 | 0.8836 | 0.8847 | 0.8587 | 0.8576 |
|  | HC vs. AD | 0.8960 | 0.8932 | 0.8910 | 0.8895 | 0.8827 | 0.8893 | 0.8807 | 0.8694 | 0.8687 |
|  | PPD vs. AD | 0.8546 | 0.8498 | 0.8213 | 0.8542 | 0.8130 | 0.8000 | 0.8269 | 0.7844 | 0.7794 |
| 10 | HC vs. PPD | 0.9126 | 0.9320 | 0.9067 | 0.9464 | 0.9266 | 0.8824 | 0.9154 | 0.8902 | 0.8952 |
|  | HC vs. AD | 0.8957 | 0.8938 | 0.8941 | 0.8922 | 0.8886 | 0.8798 | 0.8723 | 0.8665 | 0.8660 |
|  | PPD vs. AD | 0.8567 | 0.8709 | 0.8639 | 0.9125 | 0.8336 | 0.8303 | 0.8751 | 0.8339 | 0.8293 |
| 11 | HC vs. PPD | 0.9120 | 0.9058 | 0.9159 | 0.9330 | 0.8906 | 0.8982 | 0.9297 | 0.9138 | 0.9191 |
|  | HC vs. AD | 0.8966 | 0.8941 | 0.9036 | 0.8981 | 0.8847 | 0.8955 | 0.8878 | 0.8793 | 0.8825 |
|  | PPD vs. AD | 0.8581 | 0.8950 | 0.8854 | 0.9368 | 0.8712 | 0.8519 | 0.9133 | 0.8905 | 0.8837 |
| 12 | HC vs. PPD | 0.9709 | 0.9555 | 0.9656 | 0.9606 | 0.9493 | 0.9502 | 0.9562 | 0.9364 | 0.9286 |
|  | HC vs. AD | 0.8844 | 0.8831 | 0.8744 | 0.8723 | 0.8691 | 0.8566 | 0.8462 | 0.8335 | 0.8318 |
|  | PPD vs. AD | 0.9616 | 0.9574 | 0.9279 | 0.9308 | 0.9418 | 0.8981 | 0.9080 | 0.9123 | 0.8776 |
| * (1) Baseline sociodemographic-anamnestic data, (2) mood scores, (3) stress scores, (4) mood-stress  difference scores, (5) mood scores incl. change scores, (6) stress scores incl. change scores, (7) mood-stress  difference scores, (8) combination of mood and stress scores incl. change scores, (9) combination of mood,  stress and mood-stress difference scores incl. change scores.  Best performing model for the HC vs. PPD group comparison. | | | | | | | | | | |

| *Table S8: Balanced accuracies for predictive modeling incl. baseline EPDS scores, and follow-up EPDS and MPAS scores.* | | | | | | | | | | |
| --- | --- | --- | --- | --- | --- | --- | --- | --- | --- | --- |
| Week | Classifier | Feature Combination | | | | | | | | |
|  |  | 1 | 2 | 3 | 4 | 5 | 6 | 7 | 8 | 9 |
| 0 | HC vs. PPD | 0.7683 | 0.7604 | 0.7668 | 0.7591 | 0.7603 | 0.7672 | 0.7595 | 0.7241 | 0.7227 |
|  | HC vs. AD | 0.7708 | 0.7573 | 0.7584 | 0.7507 | 0.7579 | 0.7588 | 0.7513 | 0.7487 | 0.7502 |
|  | PPD vs. AD | 0.4997 | 0.5011 | 0.5956 | 0.5619 | 0.5014 | 0.5995 | 0.5642 | 0.5725 | 0.5713 |
| 1 | HC vs. PPD | 0.7689 | 0.7689 | 0.7661 | 0.7585 | 0.7643 | 0.7770 | 0.7564 | 0.7489 | 0.7491 |
|  | HC vs. AD | 0.7706 | 0.8026 | 0.7719 | 0.7938 | 0.7958 | 0.7642 | 0.7864 | 0.7904 | 0.7896 |
|  | PPD vs. AD | 0.4998 | 0.5093 | 0.5852 | 0.6447 | 0.4998 | 0.5788 | 0.6176 | 0.5469 | 0.5493 |
| 2 | HC vs. PPD | 0.7685 | 0.7589 | 0.7516 | 0.7840 | 0.7545 | 0.7315 | 0.7728 | 0.7544 | 0.7537 |
|  | HC vs. AD | 0.7711 | 0.8018 | 0.7498 | 0.7808 | 0.7944 | 0.7523 | 0.7845 | 0.7749 | 0.7738 |
|  | PPD vs. AD | 0.4995 | 0.4884 | 0.4648 | 0.4664 | 0.4714 | 0.5456 | 0.5621 | 0.5009 | 0.5012 |
| 3 | HC vs. PPD | 0.8133 | 0.8666 | 0.8183 | 0.8449 | 0.8554 | 0.7944 | 0.8198 | 0.8133 | 0.8133 |
|  | HC vs. AD | 0.9027 | 0.9119 | 0.8961 | 0.9051 | 0.9101 | 0.8915 | 0.8919 | 0.8870 | 0.8856 |
|  | PPD vs. AD | 0.4968 | 0.4775 | 0.4785 | 0.5296 | 0.5511 | 0.5316 | 0.5910 | 0.5505 | 0.5464 |
| 4 | HC vs. PPD | 0.8143 | 0.8564 | 0.8266 | 0.8499 | 0.8600 | 0.8174 | 0.8337 | 0.8273 | 0.8280 |
|  | HC vs. AD | 0.9037 | 0.9150 | 0.8969 | 0.9003 | 0.8946 | 0.8863 | 0.8885 | 0.8835 | 0.8818 |
|  | PPD vs. AD | 0.4953 | 0.5831 | 0.6260 | 0.6540 | 0.5940 | 0.6054 | 0.6310 | 0.5905 | 0.5924 |
| 5 | HC vs. PPD | 0.8140 | 0.8352 | 0.8195 | 0.8267 | 0.8377 | 0.8132 | 0.8281 | 0.8175 | 0.8167 |
|  | HC vs. AD | 0.9031 | 0.9140 | 0.9013 | 0.9019 | 0.8995 | 0.8885 | 0.8894 | 0.8884 | 0.8903 |
|  | PPD vs. AD | 0.4967 | 0.6113 | 0.6068 | 0.6540 | 0.5930 | 0.5929 | 0.6461 | 0.5826 | 0.5844 |
| 6 | HC vs. PPD | 0.9037 | 0.8852 | 0.8763 | 0.8769 | 0.8771 | 0.8701 | 0.8693 | 0.8551 | 0.8535 |
|  | HC vs. AD | 0.9212 | 0.9150 | 0.9282 | 0.9290 | 0.9019 | 0.9123 | 0.9133 | 0.9010 | 0.8984 |
|  | PPD vs. AD | 0.7026 | 0.7247 | 0.6930 | 0.7322 | 0.7178 | 0.7161 | 0.7335 | 0.6969 | 0.6956 |
| 7 | HC vs. PPD | 0.9055 | 0.8981 | 0.8884 | 0.8857 | 0.8747 | 0.8643 | 0.8675 | 0.8586 | 0.8598 |
|  | HC vs. AD | 0.9221 | 0.9145 | 0.9251 | 0.9288 | 0.9021 | 0.9069 | 0.9098 | 0.8907 | 0.8913 |
|  | PPD vs. AD | 0.7025 | 0.7226 | 0.7247 | 0.7197 | 0.7090 | 0.7173 | 0.7297 | 0.6901 | 0.6905 |
| 8 | HC vs. PPD | 0.9050 | 0.8902 | 0.8974 | 0.8901 | 0.8650 | 0.8666 | 0.8645 | 0.8413 | 0.8412 |
|  | HC vs. AD | 0.9208 | 0.9143 | 0.9179 | 0.9207 | 0.9017 | 0.9064 | 0.9077 | 0.8871 | 0.8867 |
|  | PPD vs. AD | 0.7016 | 0.7695 | 0.7555 | 0.7857 | 0.7334 | 0.7175 | 0.7538 | 0.7116 | 0.7145 |
| 9 | HC vs. PPD | 0.9051 | 0.8898 | 0.8860 | 0.8841 | 0.8817 | 0.8616 | 0.8712 | 0.8463 | 0.8455 |
|  | HC vs. AD | 0.9026 | 0.8983 | 0.9052 | 0.9002 | 0.8878 | 0.8898 | 0.8826 | 0.8686 | 0.8653 |
|  | PPD vs. AD | 0.8492 | 0.8427 | 0.8159 | 0.8498 | 0.8073 | 0.7937 | 0.8216 | 0.7843 | 0.7774 |
| 10 | HC vs. PPD | 0.9047 | 0.9118 | 0.8908 | 0.9286 | 0.9068 | 0.8560 | 0.8918 | 0.8675 | 0.8680 |
|  | HC vs. AD | 0.9019 | 0.9007 | 0.9007 | 0.8999 | 0.8909 | 0.8880 | 0.8832 | 0.8659 | 0.8643 |
|  | PPD vs. AD | 0.8487 | 0.8661 | 0.8626 | 0.9068 | 0.8305 | 0.8273 | 0.8703 | 0.8323 | 0.8274 |
| 11 | HC vs. PPD | 0.9044 | 0.8980 | 0.8874 | 0.9177 | 0.8848 | 0.8701 | 0.9220 | 0.9117 | 0.9175 |
|  | HC vs. AD | 0.9031 | 0.8994 | 0.9035 | 0.9022 | 0.8860 | 0.8910 | 0.8862 | 0.8769 | 0.8796 |
|  | PPD vs. AD | 0.8486 | 0.8893 | 0.8807 | 0.9345 | 0.8661 | 0.8493 | 0.9127 | 0.8906 | 0.8840 |
| 12 | HC vs. PPD | 0.9727 | 0.9646 | 0.9623 | 0.9630 | 0.9609 | 0.9568 | 0.9557 | 0.9452 | 0.9311 |
|  | HC vs. AD | 0.8863 | 0.8607 | 0.8613 | 0.8544 | 0.8490 | 0.8593 | 0.8507 | 0.8492 | 0.8458 |
|  | PPD vs. AD | 0.9711 | 0.9588 | 0.9373 | 0.9399 | 0.9450 | 0.9056 | 0.9187 | 0.9128 | 0.8717 |
| * (1) Baseline sociodemographic-anamnestic data, (2) mood scores, (3) stress scores, (4) mood-stress  difference scores, (5) mood scores incl. change scores, (6) stress scores incl. change scores, (7) mood-stress  difference scores, (8) combination of mood and stress scores incl. change scores, (9) combination of mood,  stress and mood-stress difference scores incl. change scores.  Best performing model for the HC vs. AD group comparison. | | | | | | | | | | |

| *Table S9: Balanced accuracies for predictive modeling without baseline* sociodemographic-*anamnestic data.* | | | | | | | | | | |
| --- | --- | --- | --- | --- | --- | --- | --- | --- | --- | --- |
| Week | Classifier | Feature Combination * | | | | | | | | |
|  |  | 1 | 2 | 3 | 4 | 5 | 6 | 7 | 8 | 9 |
| 0 | HC vs. PPD | 0.7214 | 0.5753 | 0.4650 | 0.4806 | 0.5749 | 0.4674 | 0.4785 | 0.5772 | 0.5789 |
|  | HC vs. AD | 0.7517 | 0.6423 | 0.5885 | 0.6116 | 0.6423 | 0.5895 | 0.6112 | 0.6286 | 0.6283 |
|  | PPD vs. AD | 0.4769 | 0.5010 | 0.5967 | 0.5614 | 0.5003 | 0.5969 | 0.5645 | 0.5725 | 0.5733 |
| 1 | HC vs. PPD | 0.7196 | 0.7480 | 0.6209 | 0.7364 | 0.7346 | 0.6023 | 0.7135 | 0.6922 | 0.6920 |
|  | HC vs. AD | 0.7519 | 0.8027 | 0.6904 | 0.7815 | 0.7741 | 0.6776 | 0.7785 | 0.7751 | 0.7732 |
|  | PPD vs. AD | 0.4774 | 0.5071 | 0.5861 | 0.6480 | 0.4984 | 0.5764 | 0.6186 | 0.5503 | 0.5476 |
| 2 | HC vs. PPD | 0.7187 | 0.7219 | 0.6844 | 0.7698 | 0.7171 | 0.6711 | 0.7561 | 0.7340 | 0.7332 |
|  | HC vs. AD | 0.7495 | 0.7863 | 0.6282 | 0.7939 | 0.7758 | 0.6708 | 0.7673 | 0.7550 | 0.7554 |
|  | PPD vs. AD | 0.4798 | 0.4823 | 0.4644 | 0.4698 | 0.4723 | 0.5425 | 0.5634 | 0.5008 | 0.5034 |
| 3 | HC vs. PPD | 0.7186 | 0.7812 | 0.7059 | 0.7813 | 0.7372 | 0.6942 | 0.7696 | 0.7612 | 0.7608 |
|  | HC vs. AD | 0.7516 | 0.6855 | 0.6125 | 0.6791 | 0.7707 | 0.6389 | 0.7669 | 0.7534 | 0.7541 |
|  | PPD vs. AD | 0.4755 | 0.4826 | 0.4801 | 0.5393 | 0.5570 | 0.5396 | 0.5915 | 0.5522 | 0.5472 |
| 4 | HC vs. PPD | 0.7178 | 0.7679 | 0.7373 | 0.7809 | 0.7643 | 0.7179 | 0.7754 | 0.7849 | 0.7862 |
|  | HC vs. AD | 0.7523 | 0.6515 | 0.5810 | 0.6527 | 0.6878 | 0.5854 | 0.6811 | 0.6846 | 0.6844 |
|  | PPD vs. AD | 0.4763 | 0.5960 | 0.6471 | 0.6700 | 0.6012 | 0.6114 | 0.6399 | 0.5956 | 0.5939 |
| 5 | HC vs. PPD | 0.7171 | 0.8039 | 0.6877 | 0.7867 | 0.7724 | 0.7035 | 0.7783 | 0.7689 | 0.7689 |
|  | HC vs. AD | 0.7510 | 0.6303 | 0.5267 | 0.5819 | 0.6431 | 0.5608 | 0.6545 | 0.6420 | 0.6435 |
|  | PPD vs. AD | 0.4764 | 0.6190 | 0.6154 | 0.6670 | 0.6023 | 0.5932 | 0.6502 | 0.5851 | 0.5855 |
| 6 | HC vs. PPD | 0.7200 | 0.8192 | 0.7082 | 0.8178 | 0.8065 | 0.6966 | 0.8111 | 0.8021 | 0.8013 |
|  | HC vs. AD | 0.7517 | 0.6638 | 0.5666 | 0.5582 | 0.6486 | 0.5376 | 0.5875 | 0.6236 | 0.6237 |
|  | PPD vs. AD | 0.4762 | 0.7588 | 0.6377 | 0.7535 | 0.7317 | 0.6132 | 0.7424 | 0.6967 | 0.6980 |
| 7 | HC vs. PPD | 0.7208 | 0.7846 | 0.6955 | 0.7405 | 0.7959 | 0.6883 | 0.8007 | 0.7928 | 0.7921 |
|  | HC vs. AD | 0.7506 | 0.5950 | 0.4598 | 0.5081 | 0.6487 | 0.5521 | 0.6169 | 0.6118 | 0.6120 |
|  | PPD vs. AD | 0.4755 | 0.7390 | 0.6901 | 0.7051 | 0.7330 | 0.6443 | 0.7416 | 0.6991 | 0.7029 |
| 8 | HC vs. PPD | 0.7169 | 0.8157 | 0.7211 | 0.7989 | 0.8066 | 0.6933 | 0.7735 | 0.7852 | 0.7842 |
|  | HC vs. AD | 0.7517 | 0.5677 | 0.5192 | 0.5303 | 0.6326 | 0.5464 | 0.5775 | 0.5911 | 0.5919 |
|  | PPD vs. AD | 0.4766 | 0.7949 | 0.7079 | 0.8137 | 0.7531 | 0.6507 | 0.7704 | 0.7266 | 0.7268 |
| 9 | HC vs. PPD | 0.7181 | 0.7923 | 0.7468 | 0.8209 | 0.8048 | 0.7176 | 0.8179 | 0.8103 | 0.8082 |
|  | HC vs. AD | 0.7514 | 0.4964 | 0.4702 | 0.4794 | 0.6314 | 0.5216 | 0.5939 | 0.5936 | 0.5954 |
|  | PPD vs. AD | 0.4763 | 0.7978 | 0.7396 | 0.8352 | 0.7777 | 0.6955 | 0.8319 | 0.7780 | 0.7766 |
| 10 | HC vs. PPD | 0.7186 | 0.8342 | 0.7772 | 0.8945 | 0.8175 | 0.7593 | 0.8611 | 0.8301 | 0.8289 |
|  | HC vs. AD | 0.7500 | 0.5519 | 0.4916 | 0.5325 | 0.6165 | 0.5369 | 0.5759 | 0.5798 | 0.5813 |
|  | PPD vs. AD | 0.4770 | 0.8493 | 0.7620 | 0.8985 | 0.8194 | 0.7412 | 0.8623 | 0.7988 | 0.8024 |
| 11 | HC vs. PPD | 0.7192 | 0.8795 | 0.8112 | 0.9200 | 0.8625 | 0.8066 | 0.9019 | 0.8805 | 0.8788 |
|  | HC vs. AD | 0.7512 | 0.5004 | 0.4602 | 0.4677 | 0.6172 | 0.5277 | 0.6124 | 0.5720 | 0.5732 |
|  | PPD vs. AD | 0.4760 | 0.8863 | 0.8123 | 0.9168 | 0.8612 | 0.7931 | 0.8980 | 0.8489 | 0.8573 |
| 12 | HC vs. PPD | 0.7207 | 0.8959 | 0.7668 | 0.9099 | 0.8690 | 0.7818 | 0.8817 | 0.9025 | 0.9012 |
|  | HC vs. AD | 0.7519 | 0.4737 | 0.5454 | 0.4948 | 0.5136 | 0.5009 | 0.5090 | 0.5579 | 0.5572 |
|  | PPD vs. AD | 0.4763 | 0.8950 | 0.8158 | 0.9080 | 0.8601 | 0.7821 | 0.8681 | 0.8088 | 0.8182 |
| * (1) Baseline sociodemographic-anamnestic data, (2) mood scores, (3) stress scores, (4) mood-stress  difference scores, (5) mood scores incl. change scores, (6) stress scores incl. change scores, (7) mood-stress  difference scores, (8) combination of mood and stress scores incl. change scores, (9) combination of mood,  stress and mood-stress difference scores incl. change scores.  Best performing model for the PPD vs. AD group comparison. | | | | | | | | | | |

| *Table S10: Balanced accuracies for predictive modeling incl.* sociodemographic-*anamnestic data and baseline EPDS scores.* | | | | | | | | | | |
| --- | --- | --- | --- | --- | --- | --- | --- | --- | --- | --- |
| Week | Classifier | Feature Combination * | | | | | | | | |
|  |  | 1 | 2 | 3 | 4 | 5 | 6 | 7 | 8 | 9 |
| 0 | HC vs. PPD | 0.7218 | 0.7228 | 0.7210 | 0.7216 | 0.7213 | 0.7200 | 0.7209 | 0.7211 | 0.7215 |
|  | HC vs. AD | 0.7473 | 0.7537 | 0.7483 | 0.7536 | 0.7538 | 0.7489 | 0.7544 | 0.7519 | 0.7513 |
|  | PPD vs. AD | 0.4766 | 0.4789 | 0.5530 | 0.5331 | 0.4762 | 0.5543 | 0.5305 | 0.5350 | 0.5335 |
| 1 | HC vs. PPD | 0.7196 | 0.7208 | 0.7211 | 0.7195 | 0.7215 | 0.7214 | 0.7223 | 0.7223 | 0.7211 |
|  | HC vs. AD | 0.7496 | 0.7881 | 0.7653 | 0.7913 | 0.7916 | 0.7629 | 0.7906 | 0.7845 | 0.7834 |
|  | PPD vs. AD | 0.4757 | 0.4854 | 0.5446 | 0.5887 | 0.4780 | 0.5468 | 0.5720 | 0.5211 | 0.5229 |
| 2 | HC vs. PPD | 0.7219 | 0.7210 | 0.7225 | 0.7197 | 0.7208 | 0.7224 | 0.7230 | 0.7214 | 0.7285 |
|  | HC vs. AD | 0.7499 | 0.8120 | 0.7590 | 0.7971 | 0.8053 | 0.7541 | 0.7967 | 0.7867 | 0.7861 |
|  | PPD vs. AD | 0.4772 | 0.4675 | 0.4532 | 0.4540 | 0.4556 | 0.5177 | 0.5284 | 0.4810 | 0.4848 |
| 3 | HC vs. PPD | 0.7204 | 0.7211 | 0.7211 | 0.7194 | 0.7210 | 0.7205 | 0.7204 | 0.7205 | 0.7083 |
|  | HC vs. AD | 0.7498 | 0.8123 | 0.7474 | 0.7933 | 0.8048 | 0.7537 | 0.7937 | 0.7899 | 0.7886 |
|  | PPD vs. AD | 0.4779 | 0.4683 | 0.4651 | 0.5103 | 0.5292 | 0.5129 | 0.5623 | 0.5257 | 0.5276 |
| 4 | HC vs. PPD | 0.7193 | 0.7211 | 0.7219 | 0.7217 | 0.7206 | 0.7221 | 0.7213 | 0.7212 | 0.7130 |
|  | HC vs. AD | 0.7503 | 0.7670 | 0.7438 | 0.7568 | 0.8015 | 0.7343 | 0.7781 | 0.7751 | 0.7760 |
|  | PPD vs. AD | 0.4776 | 0.5676 | 0.5914 | 0.6299 | 0.5764 | 0.5814 | 0.6131 | 0.5717 | 0.5715 |
| 5 | HC vs. PPD | 0.7201 | 0.7226 | 0.7196 | 0.7212 | 0.7217 | 0.7221 | 0.7213 | 0.7223 | 0.7122 |
|  | HC vs. AD | 0.7497 | 0.7706 | 0.7421 | 0.7561 | 0.7670 | 0.7410 | 0.7470 | 0.7580 | 0.7567 |
|  | PPD vs. AD | 0.4754 | 0.5887 | 0.5947 | 0.6382 | 0.5758 | 0.5782 | 0.6294 | 0.5724 | 0.5695 |
| 6 | HC vs. PPD | 0.7217 | 0.7232 | 0.7205 | 0.7225 | 0.7215 | 0.7187 | 0.7223 | 0.7221 | 0.7070 |
|  | HC vs. AD | 0.7485 | 0.7578 | 0.7461 | 0.7499 | 0.7638 | 0.7386 | 0.7484 | 0.7556 | 0.7557 |
|  | PPD vs. AD | 0.4787 | 0.7169 | 0.6057 | 0.7157 | 0.7020 | 0.5980 | 0.7200 | 0.6823 | 0.6810 |
| 7 | HC vs. PPD | 0.7215 | 0.7205 | 0.7226 | 0.7218 | 0.7209 | 0.7238 | 0.7187 | 0.7198 | 0.7110 |
|  | HC vs. AD | 0.7487 | 0.7475 | 0.7512 | 0.7408 | 0.7534 | 0.7442 | 0.7444 | 0.7372 | 0.7350 |
|  | PPD vs. AD | 0.4771 | 0.7100 | 0.6590 | 0.6908 | 0.7049 | 0.6244 | 0.7183 | 0.6800 | 0.6829 |
| 8 | HC vs. PPD | 0.7232 | 0.7223 | 0.7239 | 0.7216 | 0.7219 | 0.7210 | 0.7215 | 0.7219 | 0.7157 |
|  | HC vs. AD | 0.7505 | 0.7446 | 0.7422 | 0.7406 | 0.7433 | 0.7468 | 0.7399 | 0.7455 | 0.7475 |
|  | PPD vs. AD | 0.4778 | 0.7738 | 0.6785 | 0.7914 | 0.7321 | 0.6352 | 0.7518 | 0.7065 | 0.7096 |
| 9 | HC vs. PPD | 0.7211 | 0.7218 | 0.7206 | 0.7209 | 0.7200 | 0.7226 | 0.7195 | 0.7232 | 0.7136 |
|  | HC vs. AD | 0.7501 | 0.7400 | 0.7482 | 0.7434 | 0.7444 | 0.7417 | 0.7464 | 0.7343 | 0.7335 |
|  | PPD vs. AD | 0.4793 | 0.7678 | 0.7201 | 0.8206 | 0.7518 | 0.6804 | 0.8116 | 0.7583 | 0.7586 |
| 10 | HC vs. PPD | 0.7229 | 0.7221 | 0.7214 | 0.7221 | 0.7219 | 0.7212 | 0.7206 | 0.7238 | 0.7319 |
|  | HC vs. AD | 0.7496 | 0.7444 | 0.7455 | 0.7389 | 0.7469 | 0.7433 | 0.7460 | 0.7348 | 0.7350 |
|  | PPD vs. AD | 0.4753 | 0.8228 | 0.7462 | 0.8786 | 0.7930 | 0.7287 | 0.8456 | 0.7837 | 0.7904 |
| 11 | HC vs. PPD | 0.7198 | 0.7210 | 0.7231 | 0.7213 | 0.7211 | 0.7221 | 0.7215 | 0.7217 | 0.7078 |
|  | HC vs. AD | 0.7482 | 0.7430 | 0.7579 | 0.7473 | 0.7411 | 0.7473 | 0.7498 | 0.7349 | 0.7363 |
|  | PPD vs. AD | 0.4772 | 0.8776 | 0.8030 | 0.9139 | 0.8475 | 0.7796 | 0.8882 | 0.8422 | 0.8529 |
| 12 | HC vs. PPD | 0.7211 | 0.7205 | 0.7220 | 0.7217 | 0.7211 | 0.7215 | 0.7220 | 0.7209 | 0.6679 |
|  | HC vs. AD | 0.7496 | 0.7529 | 0.7526 | 0.7424 | 0.7375 | 0.7341 | 0.7234 | 0.7340 | 0.7326 |
|  | PPD vs. AD | 0.4770 | 0.8866 | 0.8053 | 0.9030 | 0.8553 | 0.7665 | 0.8693 | 0.8009 | 0.8182 |
| * (1) Baseline sociodemographic-anamnestic data, (2) mood scores, (3) stress scores, (4) mood-stress  difference scores, (5) mood scores incl. change scores, (6) stress scores incl. change scores, (7) mood-stress  difference scores, (8) combination of mood and stress scores incl. change scores, (9) combination of mood,  stress and mood-stress difference scores incl. change scores. | | | | | | | | | | |

| *Table S11: Balanced accuracies for predictive modeling incl.* sociodemographic-*anamnestic data and baseline EPDS scores, and follow-up EPDS and MPAS scores.* | | | | | | | | | | |
| --- | --- | --- | --- | --- | --- | --- | --- | --- | --- | --- |
| Week | Classifier | Feature Combination * | | | | | | | | |
|  |  | 1 | 2 | 3 | 4 | 5 | 6 | 7 | 8 | 9 |
| 0 | HC vs. PPD | 0.7215 | 0.7222 | 0.7193 | 0.7203 | 0.7209 | 0.7226 | 0.7217 | 0.7214 | 0.7211 |
|  | HC vs. AD | 0.7485 | 0.7546 | 0.7486 | 0.7563 | 0.7534 | 0.7489 | 0.7566 | 0.7503 | 0.7516 |
|  | PPD vs. AD | 0.4761 | 0.4774 | 0.5536 | 0.5304 | 0.4754 | 0.5556 | 0.5328 | 0.5387 | 0.5337 |
| 1 | HC vs. PPD | 0.7228 | 0.7192 | 0.7197 | 0.7215 | 0.7223 | 0.7209 | 0.7218 | 0.7203 | 0.7215 |
|  | HC vs. AD | 0.7492 | 0.7854 | 0.7666 | 0.7932 | 0.7900 | 0.7619 | 0.7912 | 0.7862 | 0.7839 |
|  | PPD vs. AD | 0.4758 | 0.4839 | 0.5451 | 0.5855 | 0.4788 | 0.5435 | 0.5722 | 0.5206 | 0.5202 |
| 2 | HC vs. PPD | 0.7218 | 0.7218 | 0.7212 | 0.7221 | 0.7222 | 0.7197 | 0.7198 | 0.7212 | 0.7266 |
|  | HC vs. AD | 0.7505 | 0.8099 | 0.7603 | 0.7969 | 0.8058 | 0.7531 | 0.7991 | 0.7871 | 0.7874 |
|  | PPD vs. AD | 0.4765 | 0.4675 | 0.4518 | 0.4536 | 0.4575 | 0.5201 | 0.5304 | 0.4839 | 0.4843 |
| 3 | HC vs. PPD | 0.7862 | 0.7867 | 0.7851 | 0.7861 | 0.7875 | 0.7880 | 0.7840 | 0.7854 | 0.7962 |
|  | HC vs. AD | 0.8816 | 0.8844 | 0.8721 | 0.8727 | 0.8749 | 0.8555 | 0.8588 | 0.8585 | 0.8505 |
|  | PPD vs. AD | 0.4738 | 0.4655 | 0.4630 | 0.5055 | 0.5214 | 0.5078 | 0.5606 | 0.5238 | 0.5282 |
| 4 | HC vs. PPD | 0.7859 | 0.7857 | 0.7866 | 0.7886 | 0.7867 | 0.7869 | 0.7862 | 0.7881 | 0.8022 |
|  | HC vs. AD | 0.8825 | 0.8750 | 0.8736 | 0.8705 | 0.8636 | 0.8574 | 0.8511 | 0.8484 | 0.8447 |
|  | PPD vs. AD | 0.4741 | 0.5586 | 0.5760 | 0.6171 | 0.5695 | 0.5815 | 0.6051 | 0.5708 | 0.5671 |
| 5 | HC vs. PPD | 0.7853 | 0.7860 | 0.7872 | 0.7875 | 0.7873 | 0.7871 | 0.7877 | 0.7853 | 0.7979 |
|  | HC vs. AD | 0.8805 | 0.8835 | 0.8759 | 0.8709 | 0.8658 | 0.8565 | 0.8497 | 0.8552 | 0.8516 |
|  | PPD vs. AD | 0.4737 | 0.5831 | 0.5843 | 0.6259 | 0.5697 | 0.5793 | 0.6230 | 0.5674 | 0.5706 |
| 6 | HC vs. PPD | 0.8325 | 0.8314 | 0.8316 | 0.8331 | 0.8327 | 0.8329 | 0.8315 | 0.8197 | 0.8049 |
|  | HC vs. AD | 0.8764 | 0.8723 | 0.8727 | 0.8628 | 0.8607 | 0.8614 | 0.8504 | 0.8559 | 0.8519 |
|  | PPD vs. AD | 0.6781 | 0.6950 | 0.6743 | 0.7017 | 0.6913 | 0.6981 | 0.7196 | 0.6798 | 0.6830 |
| 7 | HC vs. PPD | 0.8343 | 0.8325 | 0.8320 | 0.8329 | 0.8332 | 0.8306 | 0.8313 | 0.8199 | 0.8153 |
|  | HC vs. AD | 0.8750 | 0.8717 | 0.8699 | 0.8714 | 0.8579 | 0.8608 | 0.8491 | 0.8532 | 0.8497 |
|  | PPD vs. AD | 0.6784 | 0.6992 | 0.7013 | 0.7070 | 0.6841 | 0.6995 | 0.7133 | 0.6776 | 0.6710 |
| 8 | HC vs. PPD | 0.8320 | 0.8326 | 0.8325 | 0.8313 | 0.8327 | 0.8334 | 0.8313 | 0.8232 | 0.8125 |
|  | HC vs. AD | 0.8749 | 0.8685 | 0.8660 | 0.8687 | 0.8577 | 0.8519 | 0.8525 | 0.8493 | 0.8437 |
|  | PPD vs. AD | 0.6788 | 0.7469 | 0.7310 | 0.7638 | 0.7151 | 0.6986 | 0.7329 | 0.6959 | 0.7019 |
| 9 | HC vs. PPD | 0.8400 | 0.8383 | 0.8400 | 0.8396 | 0.8277 | 0.8398 | 0.8366 | 0.8148 | 0.8294 |
|  | HC vs. AD | 0.8604 | 0.8613 | 0.8631 | 0.8556 | 0.8471 | 0.8526 | 0.8429 | 0.8434 | 0.8387 |
|  | PPD vs. AD | 0.8209 | 0.8291 | 0.8001 | 0.8371 | 0.7901 | 0.7778 | 0.8056 | 0.7736 | 0.7697 |
| 10 | HC vs. PPD | 0.8403 | 0.8390 | 0.8401 | 0.8387 | 0.8318 | 0.8338 | 0.8390 | 0.8351 | 0.8699 |
|  | HC vs. AD | 0.8608 | 0.8586 | 0.8630 | 0.8563 | 0.8473 | 0.8507 | 0.8445 | 0.8349 | 0.8309 |
|  | PPD vs. AD | 0.8177 | 0.8490 | 0.8454 | 0.8966 | 0.8117 | 0.8176 | 0.8638 | 0.8236 | 0.8226 |
| 11 | HC vs. PPD | 0.8403 | 0.8396 | 0.8383 | 0.8390 | 0.8351 | 0.8442 | 0.8436 | 0.8474 | 0.9164 |
|  | HC vs. AD | 0.8594 | 0.8562 | 0.8673 | 0.8575 | 0.8478 | 0.8613 | 0.8511 | 0.8492 | 0.8422 |
|  | PPD vs. AD | 0.8248 | 0.8769 | 0.8696 | 0.9279 | 0.8527 | 0.8388 | 0.9075 | 0.8839 | 0.8820 |
| 12 | HC vs. PPD | 0.9669 | 0.9653 | 0.9597 | 0.9594 | 0.9608 | 0.9493 | 0.9511 | 0.9421 | 0.9250 |
|  | HC vs. AD | 0.8493 | 0.8153 | 0.8250 | 0.8034 | 0.8109 | 0.8187 | 0.8064 | 0.8134 | 0.8012 |
|  | PPD vs. AD | 0.9644 | 0.9541 | 0.9315 | 0.9346 | 0.9415 | 0.8996 | 0.9154 | 0.9109 | 0.8732 |
| * (1) Baseline sociodemographic-anamnestic data, (2) mood scores, (3) stress scores, (4) mood-stress  difference scores, (5) mood scores incl. change scores, (6) stress scores incl. change scores, (7) mood-stress  difference scores, (8) combination of mood and stress scores incl. change scores, (9) combination of mood,  stress and mood-stress difference scores incl. change scores. | | | | | | | | | | |

| *Table S12: Balanced accuracies for predictive modeling incl. follow-up MPAS scores.* | | | | | | | | | | |
| --- | --- | --- | --- | --- | --- | --- | --- | --- | --- | --- |
| Week | Classifier | Feature Combination | | | | | | | | |
|  |  | 1 | 2 | 3 | 4 | 5 | 6 | 7 | 8 | 9 |
| 0 | HC vs. PPD | 0.6505 | 0.5227 | 0.4995 | 0.4975 | 0.5251 | 0.4995 | 0.4980 | 0.5108 | 0.5774 |
|  | HC vs. AD | 0.7355 | 0.6424 | 0.5887 | 0.6116 | 0.6423 | 0.5894 | 0.6118 | 0.6289 | 0.6288 |
|  | PPD vs. AD | 0.4977 | 0.4986 | 0.5012 | 0.4961 | 0.4992 | 0.5026 | 0.4957 | 0.5952 | 0.5692 |
| 1 | HC vs. PPD | 0.6496 | 0.7485 | 0.5342 | 0.7372 | 0.7328 | 0.5894 | 0.7128 | 0.6916 | 0.6921 |
|  | HC vs. AD | 0.7352 | 0.8032 | 0.6918 | 0.7816 | 0.7747 | 0.6786 | 0.7791 | 0.7754 | 0.7732 |
|  | PPD vs. AD | 0.4978 | 0.4952 | 0.5016 | 0.5192 | 0.4576 | 0.4966 | 0.4915 | 0.5606 | 0.5498 |
| 2 | HC vs. PPD | 0.6510 | 0.7218 | 0.6850 | 0.7691 | 0.7188 | 0.6713 | 0.7562 | 0.7330 | 0.7329 |
|  | HC vs. AD | 0.7353 | 0.7866 | 0.6283 | 0.7945 | 0.7753 | 0.6712 | 0.7665 | 0.7550 | 0.7558 |
|  | PPD vs. AD | 0.4976 | 0.4987 | 0.4992 | 0.4988 | 0.4441 | 0.5278 | 0.4807 | 0.5083 | 0.4994 |
| 3 | HC vs. PPD | 0.6508 | 0.7617 | 0.6957 | 0.7704 | 0.7569 | 0.6803 | 0.7696 | 0.7739 | 0.7759 |
|  | HC vs. AD | 0.7355 | 0.7862 | 0.7336 | 0.8189 | 0.8278 | 0.7569 | 0.8145 | 0.8127 | 0.8135 |
|  | PPD vs. AD | 0.4978 | 0.4810 | 0.4760 | 0.5316 | 0.5518 | 0.5334 | 0.5874 | 0.5492 | 0.5463 |
| 4 | HC vs. PPD | 0.6517 | 0.7786 | 0.7474 | 0.7918 | 0.7699 | 0.7197 | 0.7755 | 0.7816 | 0.7820 |
|  | HC vs. AD | 0.7357 | 0.7451 | 0.7170 | 0.7369 | 0.7694 | 0.7057 | 0.7771 | 0.7640 | 0.7638 |
|  | PPD vs. AD | 0.4976 | 0.5854 | 0.6265 | 0.6543 | 0.5935 | 0.6048 | 0.6316 | 0.5889 | 0.5928 |
| 5 | HC vs. PPD | 0.6508 | 0.7883 | 0.7207 | 0.7777 | 0.7696 | 0.7182 | 0.7878 | 0.7628 | 0.7609 |
|  | HC vs. AD | 0.7358 | 0.7539 | 0.7264 | 0.7271 | 0.7408 | 0.7183 | 0.7266 | 0.7437 | 0.7426 |
|  | PPD vs. AD | 0.4986 | 0.6115 | 0.6080 | 0.6560 | 0.5919 | 0.5953 | 0.6456 | 0.5818 | 0.5847 |
| 6 | HC vs. PPD | 0.6616 | 0.8204 | 0.7033 | 0.8043 | 0.7971 | 0.6954 | 0.7941 | 0.7801 | 0.7811 |
|  | HC vs. AD | 0.7351 | 0.7656 | 0.7247 | 0.7368 | 0.7393 | 0.6963 | 0.7008 | 0.7216 | 0.7209 |
|  | PPD vs. AD | 0.4959 | 0.7449 | 0.6284 | 0.7412 | 0.7217 | 0.6126 | 0.7323 | 0.6906 | 0.6925 |
| 7 | HC vs. PPD | 0.6603 | 0.8126 | 0.7003 | 0.7629 | 0.8059 | 0.6832 | 0.7892 | 0.7829 | 0.7824 |
|  | HC vs. AD | 0.7343 | 0.7454 | 0.7316 | 0.7289 | 0.7595 | 0.7176 | 0.7229 | 0.7369 | 0.7357 |
|  | PPD vs. AD | 0.4964 | 0.7285 | 0.6816 | 0.7003 | 0.7257 | 0.6403 | 0.7344 | 0.6967 | 0.6972 |
| 8 | HC vs. PPD | 0.6612 | 0.8143 | 0.7232 | 0.7930 | 0.7933 | 0.6984 | 0.7505 | 0.7623 | 0.7627 |
|  | HC vs. AD | 0.7350 | 0.7363 | 0.7276 | 0.7289 | 0.7453 | 0.7048 | 0.7086 | 0.7210 | 0.7205 |
|  | PPD vs. AD | 0.4961 | 0.7914 | 0.6996 | 0.8106 | 0.7492 | 0.6538 | 0.7647 | 0.7213 | 0.7250 |
| 9 | HC vs. PPD | 0.6690 | 0.8192 | 0.7560 | 0.8353 | 0.8009 | 0.7371 | 0.8138 | 0.7844 | 0.7864 |
|  | HC vs. AD | 0.7239 | 0.7228 | 0.7434 | 0.7292 | 0.7167 | 0.7197 | 0.6996 | 0.7226 | 0.7226 |
|  | PPD vs. AD | 0.4918 | 0.7886 | 0.7222 | 0.8259 | 0.7691 | 0.6885 | 0.8215 | 0.7692 | 0.7660 |
| 10 | HC vs. PPD | 0.6704 | 0.8688 | 0.8089 | 0.8956 | 0.8470 | 0.7963 | 0.8595 | 0.8249 | 0.8280 |
|  | HC vs. AD | 0.7238 | 0.7234 | 0.7227 | 0.7194 | 0.7193 | 0.7225 | 0.7041 | 0.7223 | 0.7228 |
|  | PPD vs. AD | 0.4913 | 0.8436 | 0.7561 | 0.8906 | 0.8139 | 0.7338 | 0.8545 | 0.7973 | 0.7971 |
| 11 | HC vs. PPD | 0.6694 | 0.8629 | 0.8420 | 0.9079 | 0.8677 | 0.8242 | 0.8900 | 0.8649 | 0.8647 |
|  | HC vs. AD | 0.7245 | 0.7284 | 0.7226 | 0.7248 | 0.7233 | 0.7065 | 0.7200 | 0.7162 | 0.7147 |
|  | PPD vs. AD | 0.4913 | 0.8814 | 0.7992 | 0.9116 | 0.8562 | 0.7823 | 0.8941 | 0.8485 | 0.8515 |
| 12 | HC vs. PPD | 0.7135 | 0.8582 | 0.8247 | 0.8624 | 0.8548 | 0.8196 | 0.8372 | 0.8337 | 0.8333 |
|  | HC vs. AD | 0.7183 | 0.6804 | 0.6996 | 0.7010 | 0.6842 | 0.6730 | 0.6722 | 0.6765 | 0.6762 |
|  | PPD vs. AD | 0.6023 | 0.8765 | 0.8089 | 0.8944 | 0.8527 | 0.7768 | 0.8708 | 0.8137 | 0.8209 |
| * (1) Baseline sociodemographic-anamnestic data, (2) mood scores, (3) stress scores, (4) mood-stress  difference scores, (5) mood scores incl. change scores, (6) stress scores incl. change scores, (7) mood-stress  difference scores, (8) combination of mood and stress scores incl. change scores, (9) combination of mood,  stress and mood-stress difference scores incl. change scores. | | | | | | | | | | |

| *Table S13: Balanced accuracies for predictive modeling incl.* sociodemographic-*anamnestic data and baseline EPDS scores, and follow-up EPDS scores.* | | | | | | | | | | |
| --- | --- | --- | --- | --- | --- | --- | --- | --- | --- | --- |
| Week | Classifier | Feature Combination | | | | | | | | |
|  |  | 1 | 2 | 3 | 4 | 5 | 6 | 7 | 8 | 9 |
| 0 | HC vs. PPD | 0.7207 | 0.7172 | 0.7107 | 0.7024 | 0.7186 | 0.7109 | 0.7024 | 0.7107 | 0.7114 |
|  | HC vs. AD | 0.7498 | 0.7549 | 0.7485 | 0.7548 | 0.7528 | 0.7476 | 0.7535 | 0.7507 | 0.7508 |
|  | PPD vs. AD | 0.4774 | 0.4769 | 0.5577 | 0.5322 | 0.4784 | 0.5544 | 0.5335 | 0.5345 | 0.5351 |
| 1 | HC vs. PPD | 0.7206 | 0.7444 | 0.7165 | 0.7309 | 0.7374 | 0.7117 | 0.7165 | 0.7277 | 0.7279 |
|  | HC vs. AD | 0.7497 | 0.7859 | 0.7665 | 0.7924 | 0.7902 | 0.7606 | 0.7896 | 0.7856 | 0.7840 |
|  | PPD vs. AD | 0.4771 | 0.4825 | 0.5482 | 0.5910 | 0.4743 | 0.5442 | 0.5729 | 0.5227 | 0.5198 |
| 2 | HC vs. PPD | 0.7221 | 0.7545 | 0.7288 | 0.7675 | 0.7376 | 0.7139 | 0.7571 | 0.7482 | 0.7470 |
|  | HC vs. AD | 0.7495 | 0.8119 | 0.7603 | 0.7984 | 0.8077 | 0.7535 | 0.7977 | 0.7880 | 0.7882 |
|  | PPD vs. AD | 0.4758 | 0.4691 | 0.4552 | 0.4512 | 0.4548 | 0.5180 | 0.5317 | 0.4804 | 0.4840 |
| 3 | HC vs. PPD | 0.7790 | 0.8151 | 0.7764 | 0.8115 | 0.8029 | 0.7739 | 0.7976 | 0.7926 | 0.7912 |
|  | HC vs. AD | 0.8922 | 0.8976 | 0.8813 | 0.8935 | 0.8696 | 0.8620 | 0.8644 | 0.8524 | 0.8473 |
|  | PPD vs. AD | 0.4759 | 0.4697 | 0.4669 | 0.5080 | 0.5250 | 0.5101 | 0.5624 | 0.5251 | 0.5282 |
| 4 | HC vs. PPD | 0.7789 | 0.8041 | 0.7898 | 0.8152 | 0.8044 | 0.7819 | 0.8122 | 0.7957 | 0.7960 |
|  | HC vs. AD | 0.8921 | 0.8924 | 0.8727 | 0.8830 | 0.8779 | 0.8592 | 0.8682 | 0.8508 | 0.8456 |
|  | PPD vs. AD | 0.4756 | 0.5683 | 0.5909 | 0.6336 | 0.5745 | 0.5859 | 0.6135 | 0.5742 | 0.5742 |
| 5 | HC vs. PPD | 0.7782 | 0.8071 | 0.7914 | 0.8113 | 0.7967 | 0.7741 | 0.8020 | 0.7771 | 0.7778 |
|  | HC vs. AD | 0.8901 | 0.8946 | 0.8738 | 0.8840 | 0.8720 | 0.8539 | 0.8587 | 0.8505 | 0.8478 |
|  | PPD vs. AD | 0.4771 | 0.5874 | 0.5936 | 0.6319 | 0.5760 | 0.5775 | 0.6283 | 0.5722 | 0.5677 |
| 6 | HC vs. PPD | 0.8390 | 0.8386 | 0.8338 | 0.8378 | 0.8221 | 0.8246 | 0.8215 | 0.8090 | 0.8090 |
|  | HC vs. AD | 0.8864 | 0.8792 | 0.8748 | 0.8817 | 0.8646 | 0.8594 | 0.8588 | 0.8457 | 0.8408 |
|  | PPD vs. AD | 0.6879 | 0.7045 | 0.6789 | 0.7112 | 0.7043 | 0.7037 | 0.7222 | 0.6879 | 0.6863 |
| 7 | HC vs. PPD | 0.8378 | 0.8372 | 0.8338 | 0.8346 | 0.8297 | 0.8252 | 0.8253 | 0.8179 | 0.8196 |
|  | HC vs. AD | 0.8882 | 0.8773 | 0.8822 | 0.8826 | 0.8601 | 0.8582 | 0.8577 | 0.8462 | 0.8401 |
|  | PPD vs. AD | 0.6854 | 0.7032 | 0.7072 | 0.7101 | 0.6948 | 0.7034 | 0.7146 | 0.6762 | 0.6761 |
| 8 | HC vs. PPD | 0.8395 | 0.8451 | 0.8329 | 0.8427 | 0.8382 | 0.8249 | 0.8303 | 0.8259 | 0.8237 |
|  | HC vs. AD | 0.8879 | 0.8800 | 0.8792 | 0.8818 | 0.8611 | 0.8564 | 0.8606 | 0.8417 | 0.8391 |
|  | PPD vs. AD | 0.6852 | 0.7498 | 0.7321 | 0.7704 | 0.7184 | 0.7058 | 0.7382 | 0.6991 | 0.6979 |
| 9 | HC vs. PPD | 0.8496 | 0.8624 | 0.8458 | 0.8658 | 0.8398 | 0.8332 | 0.8467 | 0.8385 | 0.8363 |
|  | HC vs. AD | 0.8754 | 0.8709 | 0.8712 | 0.8724 | 0.8526 | 0.8550 | 0.8534 | 0.8391 | 0.8352 |
|  | PPD vs. AD | 0.8296 | 0.8331 | 0.8038 | 0.8407 | 0.7919 | 0.7853 | 0.8117 | 0.7754 | 0.7695 |
| 10 | HC vs. PPD | 0.8497 | 0.8815 | 0.8614 | 0.9026 | 0.8658 | 0.8395 | 0.8788 | 0.8612 | 0.8640 |
|  | HC vs. AD | 0.8794 | 0.8719 | 0.8746 | 0.8725 | 0.8534 | 0.8492 | 0.8513 | 0.8295 | 0.8246 |
|  | PPD vs. AD | 0.8290 | 0.8511 | 0.8520 | 0.9021 | 0.8162 | 0.8180 | 0.8654 | 0.8269 | 0.8221 |
| 11 | HC vs. PPD | 0.8504 | 0.8851 | 0.8724 | 0.9159 | 0.8838 | 0.8574 | 0.9159 | 0.9057 | 0.9121 |
|  | HC vs. AD | 0.8772 | 0.8688 | 0.8761 | 0.8695 | 0.8548 | 0.8580 | 0.8534 | 0.8386 | 0.8314 |
|  | PPD vs. AD | 0.8323 | 0.8798 | 0.8748 | 0.9278 | 0.8564 | 0.8425 | 0.9057 | 0.8842 | 0.8805 |
| 12 | HC vs. PPD | 0.9585 | 0.9570 | 0.9383 | 0.9455 | 0.9516 | 0.9225 | 0.9283 | 0.9265 | 0.9243 |
|  | HC vs. AD | 0.8644 | 0.7978 | 0.8017 | 0.7901 | 0.7892 | 0.7899 | 0.7850 | 0.7903 | 0.7835 |
|  | PPD vs. AD | 0.9530 | 0.9546 | 0.9185 | 0.9259 | 0.9386 | 0.8902 | 0.9029 | 0.9069 | 0.8738 |
| * (1) Baseline sociodemographic-anamnestic data, (2) mood scores, (3) stress scores, (4) mood-stress  difference scores, (5) mood scores incl. change scores, (6) stress scores incl. change scores, (7) mood-stress  difference scores, (8) combination of mood and stress scores incl. change scores, (9) combination of mood,  stress and mood-stress difference scores incl. change scores. | | | | | | | | | | |

| *Table S14: Balanced accuracies for predictive modeling incl. baseline* sociodemographic-*anamnestic data and follow-up MPAS scores.* | | | | | | | | | | |
| --- | --- | --- | --- | --- | --- | --- | --- | --- | --- | --- |
| Week | Classifier | Feature Combination | | | | | | | | |
|  |  | 1 | 2 | 3 | 4 | 5 | 6 | 7 | 8 | 9 |
| 0 | HC vs. PPD | 0.6710 | 0.6692 | 0.6530 | 0.6437 | 0.6682 | 0.6530 | 0.6445 | 0.6680 | 0.6667 |
|  | HC vs. AD | 0.7144 | 0.7261 | 0.7303 | 0.7379 | 0.7239 | 0.7306 | 0.7342 | 0.7291 | 0.7259 |
|  | PPD vs. AD | 0.4769 | 0.4792 | 0.5517 | 0.5264 | 0.4779 | 0.5564 | 0.5288 | 0.5359 | 0.5365 |
| 1 | HC vs. PPD | 0.6702 | 0.7258 | 0.6724 | 0.7010 | 0.7168 | 0.6586 | 0.6809 | 0.7080 | 0.7065 |
|  | HC vs. AD | 0.7148 | 0.7802 | 0.7336 | 0.7704 | 0.7834 | 0.7365 | 0.7771 | 0.7751 | 0.7742 |
|  | PPD vs. AD | 0.4755 | 0.4871 | 0.5484 | 0.5884 | 0.4783 | 0.5428 | 0.5733 | 0.5218 | 0.5214 |
| 2 | HC vs. PPD | 0.6724 | 0.7354 | 0.6970 | 0.7572 | 0.7162 | 0.6886 | 0.7425 | 0.7384 | 0.7381 |
|  | HC vs. AD | 0.7158 | 0.8132 | 0.7405 | 0.7932 | 0.8050 | 0.7326 | 0.7873 | 0.7849 | 0.7862 |
|  | PPD vs. AD | 0.4768 | 0.4684 | 0.4498 | 0.4555 | 0.4548 | 0.5203 | 0.5326 | 0.4835 | 0.4823 |
| 3 | HC vs. PPD | 0.6996 | 0.7648 | 0.6934 | 0.7726 | 0.7555 | 0.6967 | 0.7712 | 0.7666 | 0.7643 |
|  | HC vs. AD | 0.7717 | 0.8309 | 0.7720 | 0.8180 | 0.8319 | 0.7680 | 0.8099 | 0.8062 | 0.8049 |
|  | PPD vs. AD | 0.4734 | 0.4665 | 0.4670 | 0.5071 | 0.5220 | 0.5085 | 0.5587 | 0.5272 | 0.5255 |
| 4 | HC vs. PPD | 0.6986 | 0.7852 | 0.7064 | 0.7836 | 0.7695 | 0.6982 | 0.7798 | 0.7660 | 0.7638 |
|  | HC vs. AD | 0.7740 | 0.7880 | 0.7660 | 0.7764 | 0.8095 | 0.7524 | 0.7986 | 0.7903 | 0.7918 |
|  | PPD vs. AD | 0.4764 | 0.5571 | 0.5794 | 0.6200 | 0.5706 | 0.5815 | 0.6089 | 0.5732 | 0.5688 |
| 5 | HC vs. PPD | 0.6994 | 0.7825 | 0.7203 | 0.7944 | 0.7696 | 0.7136 | 0.7885 | 0.7623 | 0.7630 |
|  | HC vs. AD | 0.7736 | 0.8039 | 0.7686 | 0.7741 | 0.7803 | 0.7635 | 0.7654 | 0.7811 | 0.7799 |
|  | PPD vs. AD | 0.4748 | 0.5810 | 0.5885 | 0.6305 | 0.5669 | 0.5809 | 0.6249 | 0.5686 | 0.5676 |
| 6 | HC vs. PPD | 0.7088 | 0.7857 | 0.7187 | 0.7692 | 0.7730 | 0.7256 | 0.7694 | 0.7637 | 0.7651 |
|  | HC vs. AD | 0.7641 | 0.7762 | 0.7539 | 0.7568 | 0.7784 | 0.7572 | 0.7605 | 0.7706 | 0.7710 |
|  | PPD vs. AD | 0.4739 | 0.7126 | 0.5987 | 0.7101 | 0.6936 | 0.5961 | 0.7128 | 0.6776 | 0.6781 |
| 7 | HC vs. PPD | 0.7083 | 0.7806 | 0.7311 | 0.7706 | 0.7825 | 0.7261 | 0.7694 | 0.7747 | 0.7754 |
|  | HC vs. AD | 0.7650 | 0.7693 | 0.7612 | 0.7546 | 0.7644 | 0.7592 | 0.7556 | 0.7640 | 0.7617 |
|  | PPD vs. AD | 0.4720 | 0.7027 | 0.6522 | 0.6892 | 0.7004 | 0.6236 | 0.7140 | 0.6772 | 0.6796 |
| 8 | HC vs. PPD | 0.7067 | 0.7980 | 0.7379 | 0.7895 | 0.7848 | 0.7297 | 0.7741 | 0.7684 | 0.7671 |
|  | HC vs. AD | 0.7631 | 0.7576 | 0.7657 | 0.7547 | 0.7603 | 0.7606 | 0.7551 | 0.7623 | 0.7648 |
|  | PPD vs. AD | 0.4735 | 0.7684 | 0.6731 | 0.7876 | 0.7252 | 0.6343 | 0.7503 | 0.7083 | 0.7085 |
| 9 | HC vs. PPD | 0.7102 | 0.7900 | 0.7508 | 0.8005 | 0.7799 | 0.7463 | 0.7812 | 0.7639 | 0.7670 |
|  | HC vs. AD | 0.7551 | 0.7472 | 0.7779 | 0.7559 | 0.7421 | 0.7718 | 0.7611 | 0.7575 | 0.7551 |
|  | PPD vs. AD | 0.4691 | 0.7610 | 0.7072 | 0.8142 | 0.7503 | 0.6762 | 0.8052 | 0.7519 | 0.7485 |
| 10 | HC vs. PPD | 0.7102 | 0.8423 | 0.7878 | 0.8440 | 0.8198 | 0.7634 | 0.8188 | 0.7952 | 0.7936 |
|  | HC vs. AD | 0.7556 | 0.7509 | 0.7681 | 0.7474 | 0.7465 | 0.7765 | 0.7576 | 0.7649 | 0.7630 |
|  | PPD vs. AD | 0.4695 | 0.8169 | 0.7400 | 0.8709 | 0.7863 | 0.7232 | 0.8404 | 0.7814 | 0.7842 |
| 11 | HC vs. PPD | 0.7132 | 0.8521 | 0.8209 | 0.8658 | 0.8396 | 0.7910 | 0.8358 | 0.8083 | 0.8071 |
|  | HC vs. AD | 0.7549 | 0.7493 | 0.7649 | 0.7521 | 0.7475 | 0.7651 | 0.7549 | 0.7561 | 0.7592 |
|  | PPD vs. AD | 0.4708 | 0.8680 | 0.7933 | 0.9063 | 0.8408 | 0.7677 | 0.8830 | 0.8406 | 0.8499 |
| 12 | HC vs. PPD | 0.7450 | 0.8185 | 0.8152 | 0.8313 | 0.8256 | 0.7969 | 0.8068 | 0.7995 | 0.8016 |
|  | HC vs. AD | 0.7572 | 0.7061 | 0.7429 | 0.7214 | 0.7050 | 0.7294 | 0.7078 | 0.7124 | 0.7115 |
|  | PPD vs. AD | 0.5613 | 0.8702 | 0.8024 | 0.8895 | 0.8447 | 0.7680 | 0.8595 | 0.8105 | 0.8245 |
| * (1) Baseline sociodemographic-anamnestic data, (2) mood scores, (3) stress scores, (4) mood-stress  difference scores, (5) mood scores incl. change scores, (6) stress scores incl. change scores, (7) mood-stress  difference scores, (8) combination of mood and stress scores incl. change scores, (9) combination of mood,  stress and mood-stress difference scores incl. change scores. | | | | | | | | | | |
